# Up-regulation of uPARAP/Endo180 during culture activation of rat hepatic stellate cells and its presence in hepatic stellate cell lines from different species

**DOI:** 10.1186/1471-2121-10-39

**Published:** 2009-05-11

**Authors:** Seyed A Mousavi, Marita S Fønhus, Trond Berg

**Affiliations:** 1 Department of Molecular Biosciences, University of Oslo, Blindernveien 31, Blindern, N-0316 Oslo, Norway; 2 Medical Genetics Laboratory, Department of Medical Genetics, Rikshospitalet University Hospital, N-0027 Oslo, Norway

## Abstract

**Background:**

The urokinase plasminogen activator receptor associated protein (uPARAP)/Endo180 is a novel endocytic receptor that mediates collagen uptake and is implicated to play a role in physiological and pathological tissue-remodelling processes by mediating intracellular collagen degradation.

**Result:**

This study investigates the expression of uPARAP/Endo180 protein and messenger RNA in primary rat hepatic stellate cell (HSC) cultures. The results show that uPARAP/Endo180 protein is not expressed in freshly isolated HSCs or during the first few days of culture while the cells still display quiescent features. In contrast, uPARAP/Endo180 protein is expressed early during HSC activation when cells are transdifferentiated into myofibroblast-like cells. Very low levels of uPARAP/Endo180 mRNA are detectable during the first days of culture but uPARAP/Endo180 mRNA is strongly up-regulated with increasing time in culture. Moreover, endocytic uptake of denatured collagen increases as transdifferentiation proceeds over time and correlates with increased expression of uPARAP/Endo180. Finally, analysis of uPARAP/Endo180 expression in four hepatic stellate cell lines from three different species showed that all these cell lines express uPARAP/Endo180 and are able to take up denatured collagen efficiently.

**Conclusion:**

These results demonstrate that uPARAP/Endo180 expression by rat HSCs is strongly up-regulated during culture activation and identify this receptor as a feature common to culture-activated HSCs.

## Background

The urokinase plasminogen activator receptor associated protein (uPARAP)/Endo180 (also known as CD280; referred to hereafter as Endo180) is an endocytic receptor which together with the mannose receptor, the M-type phospholipase A_2 _receptor and the dendritic cell receptor DEC-205 constitutes the mannose receptor family of C-type lectins [1-3]. Endo180 was discovered as a constitutively recycling receptor of unknown function with a molecular mass of 180 kDa [4], and subsequently identified as a transcript of a gene encoding a novel member of the mannose receptor family of C-type lectins [5]. It was later recognized as a collagen-binding receptor and was referred to as urokinase plasminogen activator (uPA) receptor (uPAR) associated protein (uPARAP) because of its ability to form a ternary complex on the cell surface with uPA and its receptor uPAR [6]. Further work has established that Endo180 can act as an endocytic receptor to mediate the uptake and degradation of both native and denatured collagens through clathrin-dependent endocytosis. Endo180 may have a role in the catabolism of extracellular matrix (ECM) collagen [7-15]. Recent studies suggest that Endo180 can also exert functions beyond endocytosis of collagen including cell-matrix adhesion and cell migration [8,14,16,17].

Hepatic stellate cells (HSCs), located in the space of Disse, are the main storage site for vitamin A (in the form of retinyl ester-containing lipid droplets) in the body and they also contribute to the production of ECM proteins [18-20]. In normal liver, HSCs are essentially quiescent, but have the ability to transdifferentiate into myofibroblast-like cells in response to liver injury during a process termed "activation" [21].

Liver injury can occur as a consequence of a wide variety of causes including T cell-mediated response to viral infection, toxic metabolites from ethanol metabolism, and autoimmune hepatitis [21-24]. Activation of HSCs represents a key process in a wound healing program initiated in response to such injuries. Responding to paracrine stimuli such as transforming growth factor-β1, platelet derived growth factor, insulin-like growth factor and/or to material released from necrotic/apoptotic hepatocytes, quiescent HSCs undergo changes in gene expression, down-regulating genes involved in the preservation of the quiescent state while up-regulating genes that promote HSC myofibroblastic functions and thereby hepatic tissue remodelling. They acquire a fibrogenic phenotype leading to enhanced production and secretion of ECM components. They also produce and secrete matrix metalloproteinases (MMPs) that degrade excess of ECM and inhibitors that limit the action of MMPs. Moreover, HSCs loose their vitamin A contents and acquire myofibroblastic properties including contractility which are important for vasoregulation and wound closure [21,25-30].

Culturing of HSCs isolated from normal livers on tissue culture plastic will also cause activation of quiescent HSCs and their transdifferentiation into proliferating myofibroblastic cells, features similar to those observed in HSCs activated *in vivo *[31,32]. We have previously shown that Endo180 expressed by culture-activated rat HSCs is the main receptor in these cells mediating endocytosis of denatured collagen [13]. In this report, we used this cell culture system to investigate the expression of Endo180 in the primary culture of rat HSCs to see whether Endo180 is expressed constitutively by quiescent HSCs or if it is a component in the transdifferentiation program that is induced during the course of the activation process.

## Results

### Morphological and marker characterization of HSCs in primary culture

**Figure 1:**
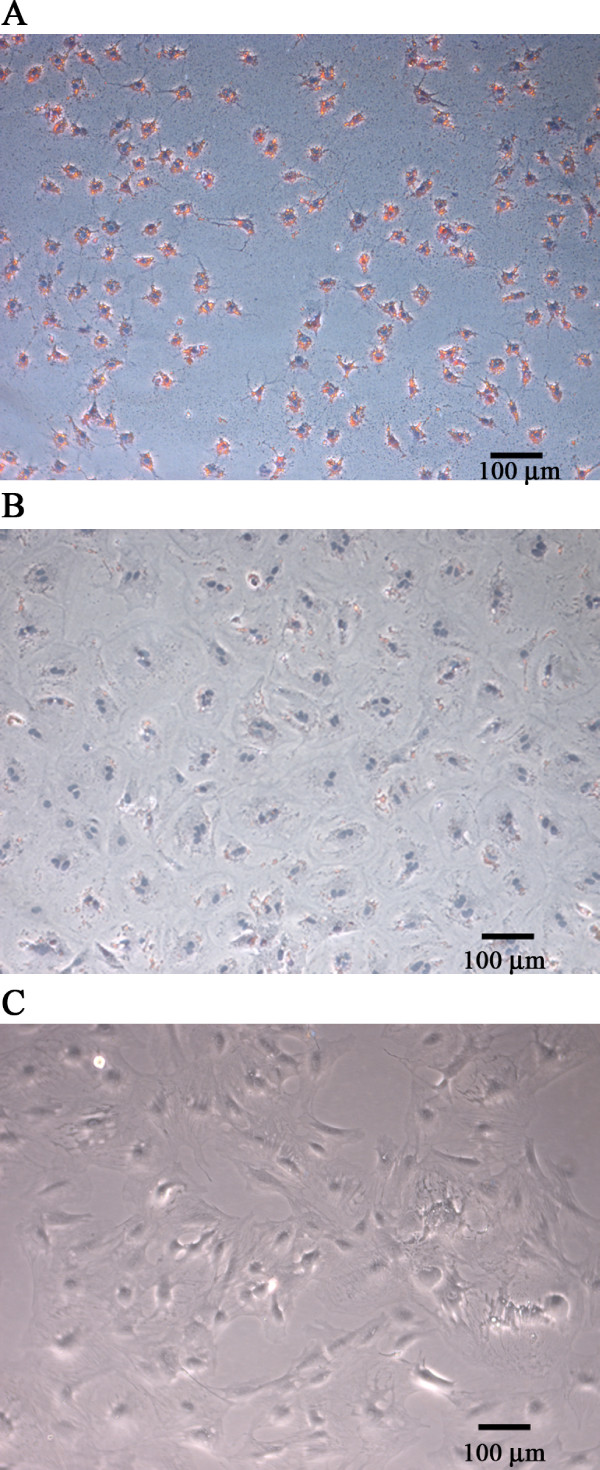
**Light microscopic analysis of HSCs stained with Oil Red-O at different stages of transdifferentiation in primary culture**. (A) Representative micrographs of HSCs cultured for 40 hours (A), 7 days (B) or sub-cultured for 5 days (C) showing changes in the morphology (from stellate shape to myofibroblast-like cells) and contents of lipid droplets. Note that HSCs cultured for 40 h contain variable amounts of lipid droplets. In sub-cultured (first passage) cells, there were no detectable lipid droplets as judged by Oil Red-O staining (data not shown). Original magnification: ×100. The micrographs shown are representative of cells obtained from at least two different preparations.

After 40 hours in primary culture, the cells showed homogeneous stellate morphology with extended long processes. Oil Red-O staining showed that more than 98% of cells possess variable amounts of lipid droplets and clear nuclei (Figure 1A). All of these features are considered as phenotypic landmarks of the majority, but not all, of quiescent HSCs [33]. After 3–4 days of culture, the cells exhibited heterogeneous morphology suggestive of activating HSCs, e.g., cells in the transition from the quiescent to activated phenotype (not shown). After 6–7 days, lipid droplets were less abundant and most cells adopted morphology typical of activated HSCs: large, spread out, polygonal morphology similar to myofibroblastic cells (Figure 1B). Most cells sub-cultured for 5 days displayed myofibroblastic appearance and were devoid of lipid droplets (Figure 1C).

**Figure 2:**
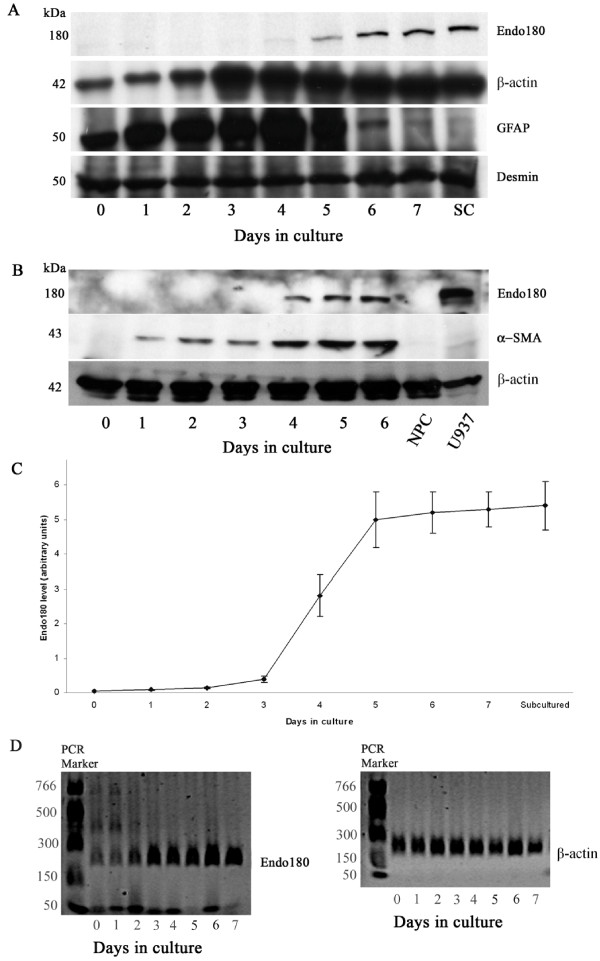
**Time course analysis of the expression of Endo180 and marker proteins in freshly isolated and cultured rat HSCs**. (A and B) Western blot analysis of expression of Endo180, desmin, GFAP, and α-SMA during culture activation of rat HSCs. At the time points shown cells were collected and cell lysates were prepared from frozen cell pellets (A) or cells were lysed directly in lysis buffer (B) and the same amount of proteins was analyzed by SDS-PAGE and Western blot using anti-Endo180 antibody. The blots were re-probed with antibodies to β-actin, used as a loading control, and marker proteins as indicated. Total non-parenchymal cells (NPC) were prepared from rat liver by collagenase perfusion as previously described [59]. (C) Densitometric analysis of Western blots showing the time-dependent increase in Endo180 protein expression in cultured rat HSCs. The ratio Endo180/β-actin bands was quantified for each day and expressed as means ± SD of seven different samples (days 1–5, and 7) or four different samples (days 0, 6 and sub-cultured). Note that there is less beta-actin in the three first lanes in Fig. 2A. However, densitometric analysis of bands showed that the amount of beta-actin at day 3 is ~ twice as much as day 7, yet no expression of Endo180 is observed at day 3. (D) RT-PCR analysis of Endo180 mRNA expression during culture activation of rat HSCs. Total RNA was extracted from cells and Endo180 and β-actin, used as an internal control, mRNAs were analyzed by RT-PCR. One representative experiment (out of three independent experiments) is shown. SC, sub-cultured (first passage).

Cultured HSCs were also characterized by the expression of quiescent and activation protein markers using Western blotting. The intermediate filament desmin is expressed in quiescent rat HSCs and is maintained during activation [34,35]. Quiescent rat HSCs also express glial fibrillary acidic protein (GFAP), another intermediate filament protein [36,37], but down-regulate this marker when they transdifferentiate into myofibroblasts [37-41]. In contrast, HSCs in the quiescent state do not express smooth muscle α-actin (α-SMA), a cytoskeletal protein important for contractile activity of myofibroblasts, but induce its expression when they acquire an activated phenotype [42,43]. In this study, we used these markers to confirm the identity of HSCs during culture activation. As expected, desmin expression was high in freshly isolated cells and significant expression was also maintained in activated state. Expression of GFAP was also high in freshly isolated and activating HSCs but was down-regulated in fully activated cells. Smooth muscle α-actin was absent from freshly isolated HSCs but its expression was rapidly induced during the first few days of culture (see Figure 2A and 2B).

### Changes in Endo180 protein and mRNA levels during rat HSC activation *in vitro*

We first examined the expression of Endo180 protein by Western blot analysis using lysates prepared from freshly isolated and cultured HSCs 1–7 days after plating. No band corresponding to Endo180 was detected in lysates from freshly isolated cells and cultured cells during the first three days. However, a band corresponding to Endo180 (approximately 180 kDa) was detectable by day 4 of culture activation, and increased approximately 2-fold in the following two days and remained unchanged thereafter (Fig 2A and 2B). In one of seven separate isolations the 180-kDa band was detected weakly in 3-day cultured HSCs. The human U937 monocytic cell line and non-parenchymal cells prepared from rat liver were used as positive and negative controls, respectively, for the expression of Endo180 (Fig 2B). No bands were detected when a non-specific rabbit immunoglobulin G was used (data not shown). To determine whether the increased Endo180 protein expression was paralleled by increase in mRNA expression, we examined the expression of Endo180 by RT-PCR of total RNA. Very low levels of Endo180 mRNA were detectable in freshly isolated HSCs and cultured cells during the first two days. However, culturing of cells for three days resulted in a strong up-regulation of Endo180 mRNA expression. Maximal up-regulation was observed between days 5 and 6 (Fig. 2D).

### Up-regulation of Endo180 correlates with increased uptake of denatured collagen

**Figure 3:**
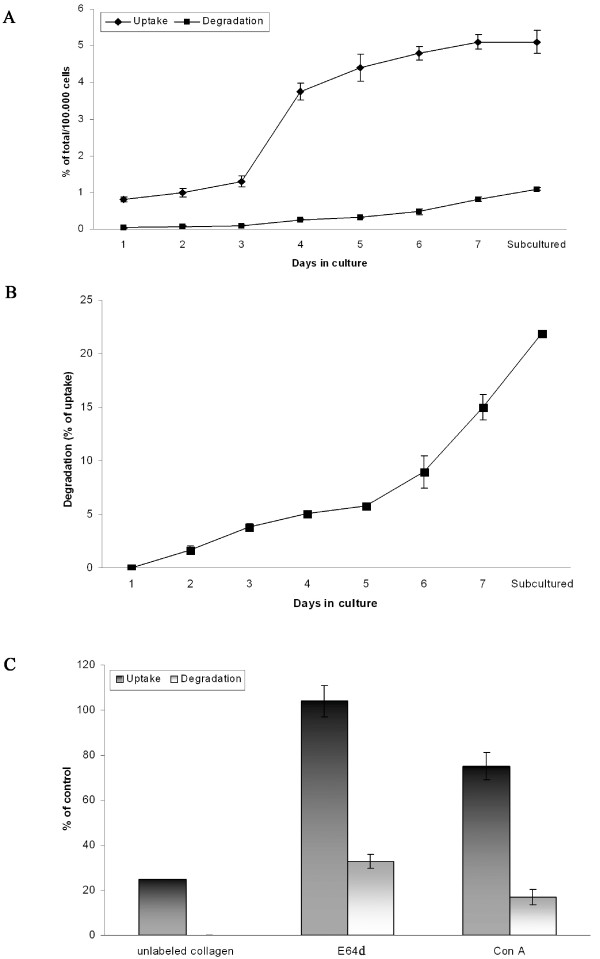
**Uptake and degradation of denatured collagen by rat HSCs as a function of cell culture time and the effect of inhibitors of lysosomal function on these processes**. (A) At each day, cells cultured in 6-well plates were washed, pre-incubation for 45 min at 37°C, and then incubated for 2 h at 37°C with 10 μg/ml ^125^I-TC-collagen. After washing, cells were lysed to determine total cell-associated radioactivity (= the sum of acid-soluble and acid-precipitable radioactivity), which was taken as a measure of total uptake of ^125^I-TC-collagen. Acid-soluble radioactivity was taken as a measure of degradation of ^125^I-TC-collagen. (B) The calculated percentage of internalized ^125^I-TC-collagen degraded by cells. Results are shown as means ± SD for triplicate wells and represent one of three similar experiments. Data are normalized for cell number. Cells were counted on the indicated days as described in Materials and methods. (C) Sub-cultured (first passage) HSCs were washed, pre-incubation for 45 min at 37°C, and then incubated at 37°C with ^125^I-TC-collagen for either 30 min in the absence or presence of excess unlabeled denatured collagen (100-fold) or for 2 h in the absence or presence of E64d (100 μM) or concanamycin A (Con A, 1.5 μM). Uptake and degradation were determined as described above. Data represent either average of duplicate determinations (unlabeled collagen) or means ± SD for triplicate determinations (E64d and Con A) and are representative for at least two such experiments performed.

We previously showed that the polyclonal anti-Endo180 antibody employed in this study is able to inhibit both cell-surface binding and endocytosis of denatured collagen by culture-activated rat HSCs [13]. To determine whether increased expression of Endo180 in cultured HSCs results in increased uptake of denatured collagen, cultured HSCs at different time points were assayed for their ability to take up and degrade denatured collagen. To this end, we used ^125^I-TC-labeling of denatured collagen, which has the advantage that the released ^125^I-TC (after degradation of ligand) is trapped in intracellular compartments and both internalization and degradation can directly be measured in cell lysates. The results showed a parallel increase in the uptake of ^125^I-TC-collagen and expression of Endo180 as the cells became more activated (Fig. 3A). Activated HSCs also showed a significant increase in their capacity to degrade the internalized ^125^I-TC-collagen from an average 5.1% (of internalized) in 4-day cells to 15% in activated cells (7-day cells) and to 22% in sub-cultured cells (Fig. 3B).

Incubation of cells in the presence of a 100-fold excess of unlabeled denatured collagen for 30 min at 37°C resulted in significant inhibition of the uptake of ^125^I-TC-collagen, indicating receptor-mediated internalization from the cell surface (Fig. 3C). The degradation of ^125^I-TC-collagen was significantly reduced in E64d-treated cells, by ~67%, indicating the involvement of cysteine proteases in the degradation of internalized ligand. The total uptake was not affected by E64d, indicating that receptor endocytic trafficking was normal (Fig. 3C). Incubating cells with concanamycin A, a drug that inhibits the activity of the vacuolar proton pump, inhibited the degradation of ^125^I-TC-collagen by ~84%, indicating that degradation of internalized ligand occurs in acidic compartments (late endosomes/lysosomes). However, the total uptake was also reduced, by ~25%, by this treatment (Fig. 3C), probably due to the continuous recycling and re-internalization of ligand-occupied receptors, which prevent them from participating in further rounds of ligand uptake [44]. Taken together, these results suggest that the up-regulation of Endo180 in rat HSCs is associated with an increased ability of cells to take up and degrade denatured collagen. Furthermore, the data suggest that culture-activation of HSCs is associated with an enhanced capacity to degrade the internalized ligand, which may reflect enhanced lysosomal activity. Alternatively, the rate of ligand delivery to lysosomes may be faster in activated rat HSCs than in non-activated HSCs.

### Human and rodent HSC lines express Endo180

To see if the expression of Endo180 in activated rat HSCs is restricted to our cell culture system or common to other culture-activated HSCs, we investigated whether Endo180 is present in four established hepatic stellate cell lines from three different species: the human LX-2 cells, the murine HSC line M1-4, and two rat HSC lines, BSC, and MFBY2.

**Figure 4:**
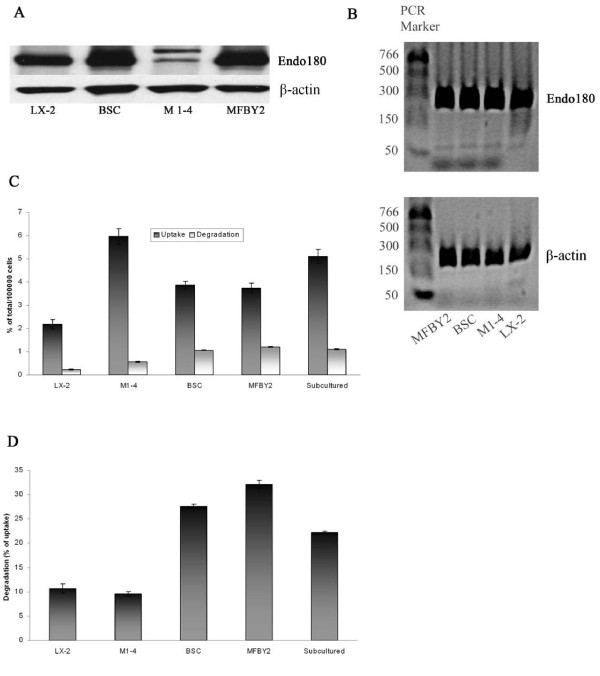
**Expression of Endo180 protein and mRNA by four established HSC lines from three different species**. Detection of Endo180 and β-actin proteins (A) and mRNAs (B) was performed as described in Figure 2. Results are representative of three (A) or two (B) experiments. (C) Uptake and degradation of denatured collagen by different HSC lines. Cells were grown in 6-well plates, washed, pre-incubation for 45 min at 37°C, and then incubated for 2 h at 37°C with 10 μg/ml ^125^I-TC-collagen. Experimental procedure is as in the legend to Figure 3. (D) The calculated percentage of internalized ^125^I-TC-collagen degraded by different HSC lines. Results are shown as means ± SD for triplicate wells and are representative for at least two similar experiments. Cells used from a new passage was treated as a separate experiment. Data are normalized for cell number.

As shown in Figure 4A and 4B, Endo180 protein and mRNA are expressed by all of these cell lines. However, the antibody against the Endo180 recognized two bands in the total cell lysate from M1-4 cells, one with a slightly higher molecular weight, probably due to different degrees of glycosylation of the Endo180 protein.

Furthermore, all four of these cell lines were able to take up and degrade ^125^I-TC-collagen (Figure 4C), although some differences in the endocytic and degradative capacities between these cell lines were noted. The murine M1-4 HSC line was more active in endocytosis and could take up approximately 1.6- and 2.7-fold more ^125^I-TC-collagen than rat and human HSC lines, respectively, during a 2-h incubation at 37°C. Despite a higher endocytic capacity, the murine M1-4 cells degraded the internalized collagen very inefficiently: only about 10% of the total internalized collagen was degraded by these cells during 2 h, which is comparable to the amount of internalized collagen degraded by the human LX-2 cells. By comparison, both rat HSC lines degraded the internalized collagen with comparable efficiencies; about 30% of the internalized, during the same incubation time (Figure 4D).

## Discussion

In this study, using primary rat HSCs, we demonstrate that (1) whereas no Endo180 protein expression was observed in freshly isolated HSCs or during the first 3 days of culture, Endo180 protein was consistently detectable after 4 days in culture, and increased further during the following days, (2) low levels of Endo180 mRNA were detectable in isolated HSCs during the first two days of culture. However, a strong up-regulation of Endo180 mRNA expression in cells after 3 days in culture was further enhanced with increasing time in culture, (3) the increase in Endo180 protein levels ran parallel with a corresponding increase in HSC uptake of denatured collagen, (4) activation of rat HSCs was associated with enhanced degradation of internalized collagen, and (5) Endo180 is expressed by both human and rodent hepatic stellate cell lines. These findings have not been described previously. We cannot, however, rule out the possibility that Endo180 mRNA might be expressed at very low levels by the majority of quiescent HSCs or at higher levels by a minor subset of quiescent HSCs.

The increase in the ability of HSCs to endocytose collagen was strikingly similar to the increase in expression of Endo180, as transdifferentiation proceeded over time. This relation between endocytic activity and receptor expression, which was also seen in established hepatic cell lines, supports earlier observations indicating that Endo180 is the main and maybe the only receptor mediating endocytosis of collagen in activated HSCs [13].

The functional importance of culture-induced up-regulation of Endo180 in the setting of liver injury is not clear at present. However, Endo180, if expressed by activated HSCs in injured livers, may have several potential functions in hepatic wound healing. The MMP-mediated cleavage of the fibrillar collagens results in the production of fragments which are rapidly denatured. Although collagen fragments thus formed can be further degraded by gelatinases [45,46], receptor-mediated endocytosis and lysosomal degradation of internalized collagen fragments by Endo180-expressing cells may also play a role in regulating the extracellular levels of collagens during tissue remodelling. Indeed, a recent study showed that collagen fragments generated by collagenase activity and heat-denatured collagen are taken up much more efficiently than intact collagen by Endo180-expressing cells [12]. Findings showing that Endo180 is highly expressed in tissues undergoing active ECM remodelling (such as during embryological development [5,47], during early postnatal bone and cartilage growth [9,48], and during gingival wound healing [49]) also points to its essential role in degradation of ECM. Moreover, several studies have shown that up-regulation of Endo180 in tumour cells [50] and stromal cells such as myofibroblasts is highly correlated with the malignancy of various carcinomas [51-53], and there is further evidence that Endo180-mediated collagen clearance accounts for tumour progression [50,51,53]. Endo180 expressed by activated HSCs may also play a role in promoting tumour invasion in the liver, given the fact that activated HSCs are a component of the stroma of hepatocellular carcinomas and can contribute to the tumour progression [54-56].

## Conclusion

The present study has identified Endo180 as a protein that is up-regulated in association with HSC activation during primary culture. These *in vitro *data suggest that Endo180 may play a role in matrix collagen turnover. In addition, Endo180 could be a good marker of culture-activated HSCs. Further investigation will be required to determine if Endo180 expression is similarly up-regulated by activated HSCs in the context of liver injury.

## Methods

### Materials

Polyclonal rabbit anti-Endo180 antibody was a gift from Dr. Clare M. Isacke (Breakthrough Breast Cancer Research Centre, The Institute of Cancer research, London, UK). This polyclonal antiserum was initially raised against purified human Endo180 [4] and we have previously shown that it also reacts with rat Endo180 [13]. Anti-desmin antibody (Cat. No. D-1033, mouse monoclonal), anti-α smooth muscle actin antibody (Cat. No. A-2547, mouse monoclonal), anti-glial fibrillary acidic protein antibody (Cat. No. G-2969, rabbit polyclonal), and anti-β-actin antibody (Cat. No. A-5316, mouse monoclonal) were obtained from Sigma. Secondary horseradish peroxidase-conjugated antibodies were obtained from GE Healthcare. Collagenase H, DNase I and protease inhibitors were obtained from Roche. Pronase E was obtained from MERCK. Culture media and Hank's balanced salt solution were from Gibco BRL (Invitrogen). Fetal bovine serum (Cat. No. A15-151) was obtained from PAA Laboratories GmbH. Antibiotics were obtained from BioWhittaker. Bovine serum albumin, collagen from calf skin (Cat. No. C-3511), and Optiprep (60% w/v) were obtained from Sigma. All other chemicals and reagents were obtained from Sigma unless otherwise specified.

### Preparation and culture of hepatic stellate cells

Animals received human care according to the guidelines of University of Oslo. Male Wistar rats, weighing 650 to 800 g, were used in experiments. HSCs were prepared by *in situ *pronase/collagenase perfusion at 37°C and density gradient centrifugation as described [57] with some modifications. Briefly, the animal was anesthetized with pentobarbital and the liver was perfused via the portal vein for about 10 min with calcium-free/magnesium-free Hank's balanced salt solution (HBSS) without phenol red. The liver was then removed and perfused with 0.35% pronase in HBSS containing calcium and magnesium and phenol red for about 20 min. This was followed by perfusion in a re-circulating system with a mixture of 0.035% collagenase and 0.1% pronase in HBSS for 20 min. Thereafter, the digested liver was suspended in HBSS containing 0.015% DNase and filtered through a sterile 100 μm nylon mash. The filtered suspension was divided in four 50-ml centrifuge tubes and centrifuged at 540 × *g *at 14°C for 7 min. The pellets were then re-suspended in HBSS containing DNase and centrifuged as described above. This was repeated once and the pellets of non-parenchymal cells were pooled and re-suspended in HBSS. The cell suspension was then mixed with 29% Optiprep solution in HBSS (to give 11.6% Optiprep), divided in two 50-ml centrifuge tubes and then overlaid with 5 ml HBSS containing 1% BSA and centrifuged at 1400 × *g *at 14°C for 23 min in a Eppendorf centrifuge (5810 R), without brake. After centrifugation, the interface layer was collected and washed twice in HBSS. After washing, the cells were re-suspended in Dulbecco's Minimum Essential medium (DMEM) containing 2 mM L-glutamine, 25 mM HEPES, 4.5 g/L glucose, penicillin (100 U/ml), streptomycin (100 μg/ml), and 18% fetal bovine serum (FBS), and counted. The cell viability was over 95% as determined by the trypan blue staining. Cells were cultured on 10 cm dishes (Costar) at a density of 3 × 10^6 ^cells/dish (for extraction of total protein and total RNA) or 6-well culture plates (Costar) at a density of 2 × 10^5 ^cells/well (for endocytosis experiments). The cells were incubated at 37°C in a humidified atmosphere containing 5% CO_2 _in air. The following day, the cells were washed twice in HBSS to remove non-adherent cells and fresh growth medium containing 18% FBS was added. One day later (day 2), the cells were washed again as above and incubated with growth medium containing 10% FBS for the next 5 days.

In some experiments, the cells were activated for 8 days in 75 cm flasks, trypsinized, recovered by centrifugation and then sub-cultured (first passage) in 10 cm dishes or 6-well plates and used after 3 to 5 days.

### Oil Red-O staining

Oil Red-O staining was used to detect cytoplasmic lipid droplets. Cells were washed with phosphate-buffered saline (PBS), pH 7.4, and fixed in 1% paraformaldehyde at room temperature for 60 min. The cells were then washed with 50% isopropanol and stained with 0.3% Oil Red-O for 10 min at room temperature, washed with PBS, and then the nuclei were stained with hematoxylin for 2 min. Finally, the cells were washed with PBS and fixed with 1% PFA before they were photographed. Microphotographs were taken using a light microscope (Leica DMIL) connected to a digital camera (Wetzler GmbH).

### Labelling of denatured collagen

Radio-labelled tyramine cellobiose (^125^I-TC) was prepared by reacting TC with Na^125^I (Perkin-Elmer) in Iodogen tubes (Pierce) and was then coupled covalently to heat-denatured collagen (5 mg in 500 μl borate buffer, pH 9.4) after activating the ^125^I-TC with the cross-linking agent cyanuric chloride, essentially as described by Pittman et al [58]. The labelled protein (^125^I-TC-collagen) was dialyzed against PBS using 10000 Da dialysis cassettes (Pierce Biotechnology) to remove non-coupled ^125^I-TC.

### Uptake and degradation experiments

Cells were washed three times with HBSS and then 1 ml DMEM containing 1% bovine serum albumin (BSA) was added. After pre-incubation for 45 min at 37°C, cells were incubated with ^125^I-TC-collagen at 37°C. For experiments where inhibitors were used, cells were either treated with inhibitor or control amounts of vehicle (DMSO) during the last 15 min of the pre-incubation period. At the end of the incubation period, the medium was removed and the cells were washed three times with ice-cold PBS, scraped in lysis buffer (0.1% SDS, 0.1 N NaOH), transferred to tubes and then precipitated with trichloracetic acid and BSA at final concentrations of 10% and 0.5%, respectively. Cell lysates were kept on ice for 15 min and centrifuged to separate acid-soluble fraction from the acid-precipitable fraction. Cell-associated acid-soluble and acid-precipitable radioactivities were measured using a γ counter. It should be noted that the ^125^I-TC moiety formed after degradation of ^125^I-TC-collagen is not released to the medium but remains trapped in degradative compartments [58]. Total uptake was calculated by subtracting radioactivity bound to the cell surface at 4°C from total cell-associated radioactivity measured at 37°C and expressed as the sum of acid-precipitable and acid-soluble radioactivity. Radioactivity bound to control wells (wells containing no cells) was less than 0.1% of the total radioactivity added to the medium. On each of days 1, 2, and 3 cells were counted directly in duplicate wells under an inverted microscope. On days 4, 5, 6, and 7 duplicate wells were trypsinized and cell number determined by use of a hemocytometer.

### Western blot analysis

At different time points, cultured HSCs were washed three times with PBS, scraped, spun down, and cell pellets were frozen at -20°C until preparation of total protein. Total protein was prepared from frozen cell pellets by addition of sample-loading buffer (Tris buffer pH 6.8, 2% Sodium dodecyl sulfate (SDS), and 10% sucrose) containing protease inhibitors. In some experiments cells were washed and lysed directly in sample-loading buffer containing protease inhibitors and stored at -20°C. Cell lysates were kept on ice for 30 min with occasional vortexing, boiled for 7 min, centrifuged, and protein concentration in the clarified lysates was determined using the BCA Protein Assay kit (Pierce). Equal amounts of protein (30–50 μg) in cell lysates were separated by 7.5% SDS-polyacrylamide gel electrophoresis (SDS-PAGE) under non-reducing conditions and electrotransferred to a polyvinylidine difluoride membrane (Millipore). The membrane was blocked in PBS containing 0.1% (v/v) Tween-20 (PBS-Tween) and 5% skim milk for 1 h at room temperature and then blotted with rabbit anti-Endo180 polyclonal antibody for 1 h. After several washes with PBS-Tween, the membrane was incubated for 1 h with horseradish peroxidase (HRP)-conjugated anti-rabbit IgG to detect bound anti-Endo180. The membrane was washed with PBS-Tween and secondary HRP-conjugated antibody was visualized by ECL detection system (GE Healthcare) and exposed to film. The membrane was stripped for re-blotting with β-actin and one or two marker proteins.

### Reverse-transcriptase polymerase chain reaction (RT-PCR)

At different time points, cultured HSCs were washed three times with PBS, scraped, spun down, and cell pellets were frozen at -20°C until preparation of total RNA. Total RNA was extracted from frozen cell pellets using Versagene RNA Cell Kit (Gentra Systems) according to the manufacturer's instruction. One microgram total RNA was reverse transcribed using SuperScript II RNase H^- ^Reverse Transcriptase (Invitrogen). After cDNA synthesis, the cDNA mixture was used as template for PCR. Routine PCR was performed using DyNAzyme (Finnzymes) and the primers (rat/mouse Endo180 forward 5'-CAC GGG AAG CCG TGT ACT AT-3' and reverse 5'-CCT CCA GGA CAG TGT GGA TT-3', human Endo180 forward 5'-GGA ACC CAA CAT CTT CCT CA-3' and reverse 5'-CCG GTC ACA CTC ATA CAT GC-3', and β-actin forward 5'-AGC CAT GTA CGT AGC CAT CC-3' and reverse 5'-TCT CAG CTG TGG TGG TGA AG-3') were designed by the Primer 3 Output program .

### Cell lines

LX-2, a human HSC line, was provided by Dr. Scott L. Friedman (Mount Sinai School of Medicine, New York). The murine M1-4 HSC line was provided by Dr. Wolfgang Mikulits (Institute of Cancer Research, Medical University of Vienna, Austria). The rat HSC lines, BSC and MFBY2, were provided by Dr. Hidekazu Tsukamoto (Keck School of Medicine, University of Southern California, Los Angeles) and Dr. Sato Kenzo (School of Life Science, Tottori University Faculty of Medicine, Japan), respectively. The murine and rat HSC lines were cultured in DMEM containing 10% FBS (Gibco BRL, Invitrogen) and kept at 37°C as described for primary cultures. LX-2 cell line was maintained under identical conditions but in Medium 199 instead of DMEM. For Western blotting and RT-PCR analyses, the cells were cultured to 80–90% confluency in 10 cm dishes and harvested as described for primary culture HSCs. For endocytosis, cells were cultured in 12-well plates at 1 × 10^5 ^cells per well twelve hours before the start of experiments and endocytosis was measured as described above. Duplicate wells were trypsinized and cell number of each cell culture was determined by a hemocytometer.

## Authors' contributions

SAM performed liver perfusion and cell preparations, carried out Western blot studies, endocytosis experiments and O-red-Oil staining and drafted the manuscript. MSF participated in the design of the study, performed RT-PCR analysis, and has been involved in drafting the manuscript. TB defined the research theme, helped to draft the manuscript and made substantial contribution to its revising. All authors read and approved the final manuscript.

## References

[B1] East L, Isacke CM (2002). The mannose receptor family. Biochimica et biophysica acta.

[B2] Llorca O (2008). Extended and bent conformations of the mannose receptor family. Cell Mol Life Sci.

[B3] Sheikh H, Yarwood H, Ashworth A, Isacke CM (2000). Endo180, an endocytic recycling glycoprotein related to the macrophage mannose receptor is expressed on fibroblasts, endothelial cells and macrophages and functions as a lectin receptor. Journal of cell science.

[B4] Isacke CM, Geer P van der, Hunter T, Trowbridge IS (1990). p180, a novel recycling transmembrane glycoprotein with restricted cell type expression. Mol Cell Biol.

[B5] Wu K, Yuan J, Lasky LA (1996). Characterization of a novel member of the macrophage mannose receptor type C lectin family. The Journal of biological chemistry.

[B6] Behrendt N, Jensen ON, Engelholm LH, Mortz E, Mann M, Dano K (2000). A urokinase receptor-associated protein with specific collagen binding properties. The Journal of biological chemistry.

[B7] East L, McCarthy A, Wienke D, Sturge J, Ashworth A, Isacke CM (2003). A targeted deletion in the endocytic receptor gene Endo180 results in a defect in collagen uptake. EMBO reports.

[B8] Engelholm LH, List K, Netzel-Arnett S, Cukierman E, Mitola DJ, Aaronson H, Kjoller L, Larsen JK, Yamada KM, Strickland DK (2003). uPARAP/Endo180 is essential for cellular uptake of collagen and promotes fibroblast collagen adhesion. The Journal of cell biology.

[B9] Howard MJ, Chambers MG, Mason RM, Isacke CM (2004). Distribution of Endo180 receptor and ligand in developing articular cartilage. Osteoarthritis and cartilage/OARS, Osteoarthritis Research Society.

[B10] Howard MJ, Isacke CM (2002). The C-type lectin receptor Endo180 displays internalization and recycling properties distinct from other members of the mannose receptor family. The Journal of biological chemistry.

[B11] Kjoller L, Engelholm LH, Hoyer-Hansen M, Dano K, Bugge TH, Behrendt N (2004). uPARAP/endo180 directs lysosomal delivery and degradation of collagen IV. Experimental cell research.

[B12] Madsen DH, Engelholm LH, Ingvarsen S, Hillig T, Wagenaar-Miller RA, Kjoller L, Gardsvoll H, Hoyer-Hansen G, Holmbeck K, Bugge TH (2007). Extracellular collagenases and the endocytic receptor, urokinase plasminogen activator receptor-associated protein/Endo180, cooperate in fibroblast-mediated collagen degradation. The Journal of biological chemistry.

[B13] Mousavi SA, Sato M, Sporstol M, Smedsrod B, Berg T, Kojima N, Senoo H (2005). Uptake of denatured collagen into hepatic stellate cells: evidence for the involvement of urokinase plasminogen activator receptor-associated protein/Endo180. The Biochemical journal.

[B14] Sturge J, Wienke D, Isacke CM (2006). Endosomes generate localized Rho-ROCK-MLC2-based contractile signals via Endo180 to promote adhesion disassembly. The Journal of cell biology.

[B15] Wienke D, MacFadyen JR, Isacke CM (2003). Identification and characterization of the endocytic transmembrane glycoprotein Endo180 as a novel collagen receptor. Molecular biology of the cell.

[B16] Sturge J, Wienke D, East L, Jones GE, Isacke CM (2003). GPI-anchored uPAR requires Endo180 for rapid directional sensing during chemotaxis. The Journal of cell biology.

[B17] Thomas EK, Nakamura M, Wienke D, Isacke CM, Pozzi A, Liang P (2005). Endo180 binds to the C-terminal region of type I collagen. The Journal of biological chemistry.

[B18] Blomhoff R, Green MH, Berg T, Norum KR (1990). Transport and storage of vitamin A. Science (New York, NY).

[B19] Geerts A (2001). History, heterogeneity, developmental biology, and functions of quiescent hepatic stellate cells. Seminars in liver disease.

[B20] Wake K (1980). Perisinusoidal stellate cells (fat-storing cells, interstitial cells, lipocytes), their related structure in and around the liver sinusoids, and vitamin A-storing cells in extrahepatic organs. International review of cytology.

[B21] Friedman SL (2003). Liver fibrosis – from bench to bedside. Journal of hepatology.

[B22] Czaja AJ (2003). Autoimmune liver disease. Current opinion in gastroenterology.

[B23] Haber PS, Warner R, Seth D, Gorrell MD, McCaughan GW (2003). Pathogenesis and management of alcoholic hepatitis. Journal of gastroenterology and hepatology.

[B24] Rehermann B, Nascimbeni M (2005). Immunology of hepatitis B virus and hepatitis C virus infection. Nature reviews.

[B25] Bataller R, Brenner DA (2005). Liver fibrosis. The Journal of clinical investigation.

[B26] Cassiman D, Roskams T (2002). Beauty is in the eye of the beholder: emerging concepts and pitfalls in hepatic stellate cell research. Journal of hepatology.

[B27] Friedman SL (2004). Mechanisms of disease: Mechanisms of hepatic fibrosis and therapeutic implications. Nature clinical practice.

[B28] Gressner AM, Bachem MG (1995). Molecular mechanisms of liver fibrogenesis – a homage to the role of activated fat-storing cells. Digestion.

[B29] Iredale JP (2001). Hepatic stellate cell behavior during resolution of liver injury. Seminars in liver disease.

[B30] Saile B, Ramadori G (2007). Inflammation, damage repair and liver fibrosis – role of cytokines and different cell types. Zeitschrift fur Gastroenterologie.

[B31] Friedman SL, Roll FJ (1987). Isolation and culture of hepatic lipocytes, Kupffer cells, and sinusoidal endothelial cells by density gradient centrifugation with Stractan. Analytical biochemistry.

[B32] Herrmann J, Gressner AM, Weiskirchen R (2007). Immortal hepatic stellate cell lines: useful tools to study hepatic stellate cell biology and function?. Journal of cellular and molecular medicine.

[B33] Wake K, Sato T (1993). Intralobular heterogeneity of perisinusoidal stellate cells in porcine liver. Cell and tissue research.

[B34] Ogawa K, Suzuki J, Mukai H, Mori M (1986). Sequential changes of extracellular matrix and proliferation of Ito cells with enhanced expression of desmin and actin in focal hepatic injury. The American journal of pathology.

[B35] Yokoi Y, Namihisa T, Kuroda H, Komatsu I, Miyazaki A, Watanabe S, Usui K (1984). Immunocytochemical detection of desmin in fat-storing cells (Ito cells). Hepatology (Baltimore, Md).

[B36] Gard AL, White FP, Dutton GR (1985). Extra-neural glial fibrillary acidic protein (GFAP) immunoreactivity in perisinusoidal stellate cells of rat liver. Journal of neuroimmunology.

[B37] Neubauer K, Knittel T, Aurisch S, Fellmer P, Ramadori G (1996). Glial fibrillary acidic protein – a cell type specific marker for Ito cells in vivo and in vitro. Journal of hepatology.

[B38] Buniatian GH (2003). Stages of activation of hepatic stellate cells: effects of ellagic acid, an inhibiter of liver fibrosis, on their differentiation in culture. Cell proliferation.

[B39] Knittel T, Aurisch S, Neubauer K, Eichhorst S, Ramadori G (1996). Cell-type-specific expression of neural cell adhesion molecule (N-CAM) in Ito cells of rat liver. Up-regulation during in vitro activation and in hepatic tissue repair. The American journal of pathology.

[B40] Niki T, De Bleser PJ, Xu G, Berg K Van Den, Wisse E, Geerts A (1996). Comparison of glial fibrillary acidic protein and desmin staining in normal and CCl4-induced fibrotic rat livers. Hepatology (Baltimore, Md.

[B41] Reynaert H, Rombouts K, Jia Y, Urbain D, Chatterjee N, Uyama N, Geerts A (2005). Somatostatin at nanomolar concentration reduces collagen I and III synthesis by, but not proliferation of activated rat hepatic stellate cells. British journal of pharmacology.

[B42] Ramadori G, Veit T, Schwogler S, Dienes HP, Knittel T, Rieder H, Meyer zum Buschenfelde KH (1990). Expression of the gene of the alpha-smooth muscle-actin isoform in rat liver and in rat fat-storing (ITO) cells. Virchows Archiv.

[B43] Rockey DC, Boyles JK, Gabbiani G, Friedman SL (1992). Rat hepatic lipocytes express smooth muscle actin upon activation in vivo and in culture. Journal of submicroscopic cytology and pathology.

[B44] Mousavi SA, Kjeken R, Berg TO, Seglen PO, Berg T, Brech A (2001). Effects of inhibitors of the vacuolar proton pump on hepatic heterophagy and autophagy. Biochimica et biophysica acta.

[B45] Matrisian LM (1992). The matrix-degrading metalloproteinases. Bioessays.

[B46] Vogel WF (2001). Collagen-receptor signaling in health and disease. Eur J Dermatol.

[B47] Engelholm LH, Nielsen BS, Netzel-Arnett S, Solberg H, Chen XD, Lopez Garcia JM, Lopez-Otin C, Young MF, Birkedal-Hansen H, Dano K (2001). The urokinase plasminogen activator receptor-associated protein/endo180 is coexpressed with its interaction partners urokinase plasminogen activator receptor and matrix metalloprotease-13 during osteogenesis. Laboratory investigation; a journal of technical methods and pathology.

[B48] Wagenaar-Miller RA, Engelholm LH, Gavard J, Yamada SS, Gutkind JS, Behrendt N, Bugge TH, Holmbeck K (2007). Complementary roles of intracellular and pericellular collagen degradation pathways in vivo. Molecular and cellular biology.

[B49] Honardoust HA, Jiang G, Koivisto L, Wienke D, Isacke CM, Larjava H, Hakkinen L (2006). Expression of Endo180 is spatially and temporally regulated during wound healing. Histopathology.

[B50] Wienke D, Davies GC, Johnson DA, Sturge J, Lambros MB, Savage K, Elsheikh SE, Green AR, Ellis IO, Robertson D (2007). The collagen receptor Endo180 (CD280) Is expressed on basal-like breast tumour cells and promotes tumour growth in vivo. Cancer research.

[B51] Curino AC, Engelholm LH, Yamada SS, Holmbeck K, Lund LR, Molinolo AA, Behrendt N, Nielsen BS, Bugge TH (2005). Intracellular collagen degradation mediated by uPARAP/Endo180 is a major pathway of extracellular matrix turnover during malignancy. The Journal of cell biology.

[B52] Schnack Nielsen B, Rank F, Engelholm LH, Holm A, Dano K, Behrendt N (2002). Urokinase receptor-associated protein (uPARAP) is expressed in connection with malignant as well as benign lesions of the human breast and occurs in specific populations of stromal cells. International journal of cancer.

[B53] Sulek J, Wagenaar-Miller RA, Shireman J, Molinolo A, Madsen DH, Engelholm LH, Behrendt N, Bugge TH (2007). Increased expression of the collagen internalization receptor uPARAP/Endo180 in the stroma of head and neck cancer. J Histochem Cytochem.

[B54] Enzan H, Himeno H, Iwamura S, Onishi S, Saibara T, Yamamoto Y, Hara H (1994). Alpha-smooth muscle actin-positive perisinusoidal stromal cells in human hepatocellular carcinoma. Hepatology (Baltimore, Md).

[B55] Johnson SJ, Burr AW, Toole K, Dack CL, Mathew J, Burt AD (1998). Macrophage and hepatic stellate cell responses during experimental hepatocarcinogenesis. Journal of gastroenterology and hepatology.

[B56] Terada T, Makimoto K, Terayama N, Suzuki Y, Nakanuma Y (1996). Alpha-smooth muscle actin-positive stromal cells in cholangiocarcinomas, hepatocellular carcinomas and metastatic liver carcinomas. Journal of hepatology.

[B57] Weiskirchen R, Gressner AM (2005). Isolation and culture of hepatic stellate cells. Methods in molecular medicine.

[B58] Pittman RC, Carew TE, Glass CK, Green SR, Taylor CA, Attie AD (1983). A radioiodinated, intracellularly trapped ligand for determining the sites of plasma protein degradation in vivo. The Biochemical journal.

[B59] Mousavi SA, Sporstol M, Fladeby C, Kjeken R, Barois N, Berg T (2007). Receptor-mediated endocytosis of immune complexes in rat liver sinusoidal endothelial cells is mediated by FcgammaRIIb2. Hepatology (Baltimore, Md).

